# Commentary on “A preoperative nomogram and web-based clinical decision support system for predicting early renal function after living donor kidney transplantation”

**DOI:** 10.1097/JS9.0000000000004670

**Published:** 2026-01-21

**Authors:** Huiwen Luo, Yaodong Wang, Haili Long

**Affiliations:** a NHC Key Laboratory of Nuclear Technology Medical Transformation, Mianyang Central Hospital, School of Medicine, University of Electronic Science and Technology of China, Mianyang, China; b Department of Urology, Mianyang Central Hospital, School of Medicine, University of Electronic Science and Technology of China, Mianyang, China

The study by Ahn *et al* represents an important advancement in pre-transplant risk assessment by introducing a fully preoperative model to predict early renal function following living donor kidney transplantation (LDKT). Unlike previous models that rely heavily on postoperative variables – such as early graft function, rejection episodes, or dynamic serum creatinine changes – this work focuses exclusively on preoperative donor and recipient characteristics, aligning prediction methodology with true clinical decision-making needs^[1,2]^.

## Major strengths

1. Large, multicenter cohort with rigorous validation

Utilizing 3335 LDKT recipients from three transplant centers over more than a decade, the study demonstrates strong internal and external validity. The model maintained stable performance even when applied to a validation cohort with substantially different immunologic and demographic characteristics, reinforcing its generalizability – an outcome consistent with recent evidence suggesting that large-scale, heterogeneous cohorts strengthen prediction stability in kidney transplantation^[3]^.

2. Exclusive use of preoperative variables enhances clinical relevance

By predicting the lowest serum creatinine within 1 year after transplantation using only preoperative information, the model aligns directly with clinical decision-making contexts such as donor-recipient matching and perioperative planning. This approach parallels the growing emphasis on anticipatory, preoperative risk stratification in transplant medicine^[4]^.

3. Donor kidney volume emerges as a key predictive biomarker

Donor kidney volume ranked among the most influential predictors. This finding is in line with recent imaging and AI-based studies demonstrating strong correlations between nephron mass and post-transplant renal function^[5]^. Given the increasing interest in quantitative imaging biomarkers, this reinforces the clinical utility of incorporating standardized kidney volume assessment.

4. Interpretability of the Basic-Parameter Model

The authors’ comparison of multiple machine learning models – including linear regression, elastic net, random forest, and gradient boosting – revealed no major advantage of complex algorithms over simpler linear models. This observation is consistent with other transplant prediction studies showing that interpretable models often match or exceed machine learning performance^[6]^. The parsimonious five-variable model enhances clinical usability without compromising accuracy.

5. Practical implementation through a nomogram and web-based CDSS

The translation of the model into a nomogram and web-based tool significantly increases clinical accessibility. Such tools are increasingly recognized as essential for integrating predictive analytics into real-world transplant workflows^[7]^.

## Points for critical consideration

1. Limited international generalizability

Although multicenter, the study population is confined to Korea. Variability in donor selection criteria, immunosuppression regimens, and demographic patterns across different countries may affect the model’s external validity. Multi-national validation aligned with recent global prediction model development standards is warranted^[8]^.

2. No integration of postoperative determinants of renal recovery

The prediction target – a postoperative nadir serum creatinine – is influenced by factors such as acute rejection, delayed graft function, perioperative hemodynamics, and immunosuppressive toxicity. While the authors’ focus on preoperative variables is intentional, hybrid models integrating dynamic postoperative data may further improve prognostic accuracy^[9]^.

3. Dependence on CT-based kidney volume measurement

Although kidney volume is highly predictive, not all transplant centers routinely perform AI-assisted segmentation. This may limit feasibility in lower-resource settings. As highlighted in recent studies, imaging standardization remains a major barrier to widespread adoption of radiomics-based predictors^[5]^.

4. Model performance sensitivity to extreme cases

The root mean square error exceeding the mean absolute error suggests that outlier cases – such as elderly donors, marginal donor kidneys, or highly sensitized recipients – may be poorly captured. Tailored sub-models for high-risk groups, as explored in contemporary studies, may enhance prediction robustness^[3,10]^.

## Clinical and research implications

This study meaningfully contributes to the movement toward precision transplant medicine by demonstrating that simple, accessible preoperative variables can provide robust prognostic insight. The model supports improved recipient selection, donor matching, preoperative counseling, and individualized postoperative monitoring intensity.

## Future research should explore

1. Integration of imaging radiomics, genomics, and immune profiling to enhance prediction accuracy.
2. Development of hybrid preoperative-plus-dynamic postoperative models.
3. Embedding the model within electronic clinical systems to enable real-time decision support.
4. Prospective evaluation of whether the model measurably improves clinical outcomes and resource utilization.

## Conclusion

Ahn *et al* provide a well-designed, clinically meaningful, and externally validated prediction model for early renal function after LDKT. The simplicity of the Basic-Parameter Model, coupled with its strong performance and practical implementation through a web-based CDSS, marks an important step toward personalized transplant care. This work enriches the growing literature promoting data-driven, preoperative decision support in kidney transplantation.

## Data Availability

All data analyzed or generated during this study are included in the article.
